# On the Mechanical Behaviour of Biomimetic Cornstalk-Inspired Lightweight Structures

**DOI:** 10.3390/biomimetics8010092

**Published:** 2023-02-24

**Authors:** Shakib Hyder Siddique, Paul J. Hazell, Gerald G. Pereira, Hongxu Wang, Juan P. Escobedo, Ali A. H. Ameri

**Affiliations:** 1 School of Engineering and Information Technology, The University of New South Wales, Canberra, ACT 2600, Australia; 2 CSIRO Data61, Private Bag 10, Clayton South, VIC 3169, Australia

**Keywords:** bioinspired design, porous structures, energy absorption, collapse mechanisms, lightweight structures

## Abstract

This paper presents an investigation on the stiffness and energy absorption capabilities of three proposed biomimetic structures based on the internal architecture of a cornstalk. 3D printing was used to manufacture specimens using a tough and impact-resistant thermoplastic material, acrylonitrile butadiene styrene (ABS). The structural stiffness, maximum stress, densification strain, and energy absorption were extracted from the compression tests performed at a strain rate of 10^−3^ s^−1^. A numerical model was developed to analyse the behaviour of the biomimetic structures under compression loading. Further, a damage examination was conducted through optical microscopy and profilometry. The results showed that the cornstalk-inspired biomimetic structure exhibited a superior specific energy absorption (SEA) capability that was three times higher than that of the other core designs as reported in the literature.

## 1. Introduction

Porous structures exist in biological materials, such as the wall of a pomelo, a lotus root, a turtle shell carapace, and bone [1,2,3,4]. The ability to improve engineering structures based on lessons from the structure of these biological materials has been made possible through recent developments in computational, manufacturing, and experimental methodologies. Biomimicry is an approach of taking inspiration from structures in nature that have desirable characteristics. Researchers tend to mimic structural features based on any one of the three scales (the macro, meso, or micro level) when designing new structures. It is a potential way to study the principles and functionalities found in biological systems to design novel structures with superior mechanical or functional properties. Prior efforts mainly focused on improving the strength, stiffness, and toughness of materials and structures [5,6,7,8]. The ability of porous biological materials to absorb mechanical energy has been studied over the past few years [9,10,11,12,13,14,15,16]. Some examples of sources of inspiration for studying porous biomimetic structures are luffas [11], pomelos [12], lotus roots [9], turtles [10], and bone [13].

Most plant stems are lightweight biological composite materials with superior stiffness-to-mass ratios compared to those of metallic materials such as steel and aluminium [17,18,19]. In nature, the stems of plants can endure high stress from axial loading. As a result, some plant stems have a distinctive structure consisting of a tubular column with regular nodes. These nodes are characterised by an internal diaphragm and an external ridge. The nodes strengthen the ability of the stem to withstand compression from the body’s weight, the hanging fruits surrounding the stem, external compression forces such as heavy rainfall, and the varying sizes of the branches while avoiding stem cracking. Each characteristic of a plant stem can be mimicked to design thin-walled structures with significant energy absorption capabilities [20].

Cornstalks are thick and strong structures [21]. They can endure mechanical stressors. Agricultural crops endure two kinds of mechanical stressors. The stress can be caused by human activity using agricultural machinery and other tools used in agricultural processes, such as pruning, transplanting, or thinning. The other form of stress is provoked by adverse weather conditions, such as withstanding impacts from the stones of a hailstorm. These activities and occurrences are prime examples of how cornstalks and other plants can endure drastic external forces [22,23,24]. 

The stem of a plant and thin-walled engineering structures have many similarities: (a) both can be tubular structures or filled structures, (b) both absorb energy during deformation, and (c) both are subjected to static and dynamic loads. One of the key characteristics of agricultural crops is their loading resistance. It is also suggested that the combination of the light weight and high strength of the stem structure provides a natural advantage. Therefore, this can provide us with new ideas for the design of energy-absorbing structures.

**Figure 1:**
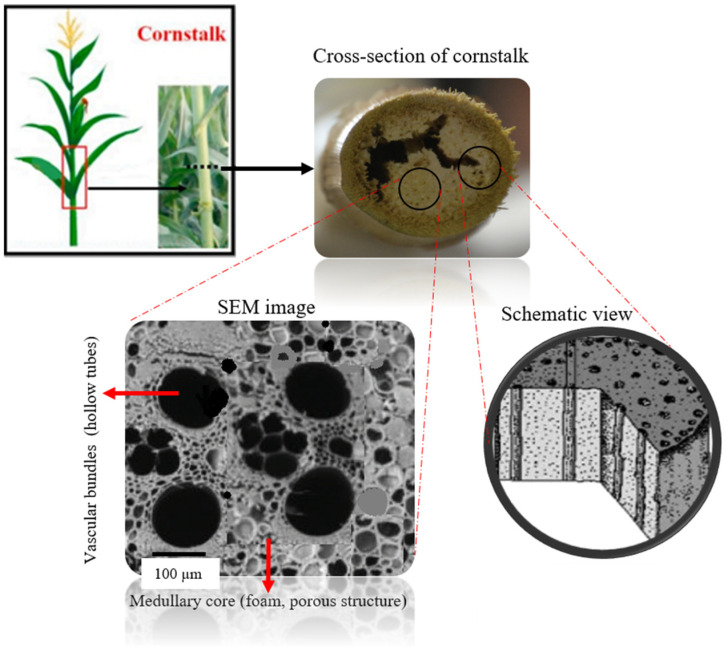
Schematic diagram illustrating the structural hierarchy of a cornstalk [20,25].

Cornstalks resemble a composite filling structure. The inner medullary core of its stem is a foam porous structure with a similar function to that of a foam core, significantly so when it can sustain its weight or other compressive forces [25]. The cross-section of a cornstalk is shown in Figure 1, and it is made up of large vascular bundles (used to carry water and nutrients) surrounded by foam matrix tissues. In addition to strengthening the support of the stem, the composition of the vascular bundles and foam matrix can also significantly lower the mass of the structure due to the presence of voids distributed around the structures.

Many scholars have investigated the properties of cornstalks themselves. For example, He and Wang [26] analysed the correlation between the fibre morphology and the tensile property of cornstalks to offer a theoretical understanding of physiochemical property research. Liu et al. [27] studied the physical properties of a cornstalk by investigating the effects of loading conditions on cracks occurring in the cornstalk. As loading resistance is one of the essential factors in the survivability of crops, Chen et al. [28] and Yuming et al. [29] investigated the loading resistance characteristics of a cornstalk. Yu et al. [30] investigated the effect of the water content in different sections of a cornstalk and its effect on the cornstalk’s mechanical properties. To reduce cutting force and power consumption and to improve cutting properties, researchers have conducted impact tests on cornstalks [24,31]. This also provides the basis for designing agricultural machinery [32] and creates a numerical model of cornstalks for simulation purposes [31]. Lastly, Robertson et al. [25] and Zhang et al. [33] investigated cornstalks’ strength and compressive behaviour to determine their load-bearing capability and their resistance to external forces.

Based on the principle of engineering mechanics, biomimetic structures are inspired by studying the architecture of organisms at different structural length scales. Song et al. [17] mimicked the cross-sectional shape of a cornstalk itself and designed bioinspired tubes. No microscopy work was needed as it was the shape of the stalk that the authors were interested in for designing the tubes. Thus, the authors mimicked a feature of the cornstalk on a macro scale to design four kinds of lightweight bionic foams enclosed in a tube made from carbon fibre and aluminium to study its energy absorption capability. The results of the bionic-foam-filled tubes demonstrated a reduction in the crushing force by 30% on the bionic designs under axial dynamic impact conditions compared to that under quasistatic loading conditions. The specific energy absorption (SEA) values of the bionic designs with carbon fibre tubes were 23% higher than those of the aluminium tubes. Based on the experimental tests, it was concluded that the bionic designs had an SEA improvement of 9.9% compared to the ordinary foam-filled tube after considering the mass reduction in the samples. 

Due to the porous architecture of biomimetic structures, their smart usage could greatly influence fuel consumption in the automotive industry due to higher fuel efficiency and a lower carbon footprint. In the case of electric vehicles, reducing the weight of the vehicle could potentially increase the driving range powered by batteries. Some examples include a diesel piston, a fluid pump, and a racing car cylinder head [34]. In all these cases, they reduce the mass of the structure while fulfilling the mechanical performance requirements. Biomimetic approaches towards lightweight reinforced composite structures for car interior parts were proposed by a few authors [35,36,37]. For rigid and lightweight frameworks, porous sandwich structures are also needed for the aerospace industry. Wings must withstand aerodynamic forces and potential bird impacts. This section comprises the outer skin, interior passageways, and feeding tubes. The outer layer of the sandwich panel also acts as the aerodynamic surface, providing protective functionalities [34,38,39,40].

This paper investigated a novel bioinspired cornstalk structure that was mimicked on the meso scale and that was subjected to compression. We present the energy absorption capability results and analysed some failure mechanisms identified through structural deformation. These objectives were achieved by employing a numerical model coupled with experimental approaches. The effect of the geometry of the biomimetic structure on its energy absorption capability was investigated. We also present an understanding of how the biomimetic-designed structures performed comparatively with other porous structures in a literature review. To mimic the tough and impact-resistant characteristics of a stem, acrylonitrile butadiene styrene (ABS)—an oil-based thermoplastic—was chosen for the three-dimensional (3D) printing of the specimens. The geometrical design and the computer-aided design (CAD) models were developed for the printing of the specimens.

## 2. Biomimetic Designs and Materials

This study investigated the energy absorption capabilities of a cornstalk on a meso scale with respect to its building blocks and was designed by replicating the vascular bundles and medullary core, which can be seen under a microscope. It was not aimed to study the entire hierarchical structure of the stem, as replicating all the stem’s structural features and mechanics may require several techniques at different length scales. Simplified models of a stem-like structure were designed representing reinforcement with cylinders embedded in a porous matrix. The authors paid attention to keeping the designed structures at a constant volume ratio near 60%, which is similar to that of the structures found in plants [41]. 

**Figure 2:**
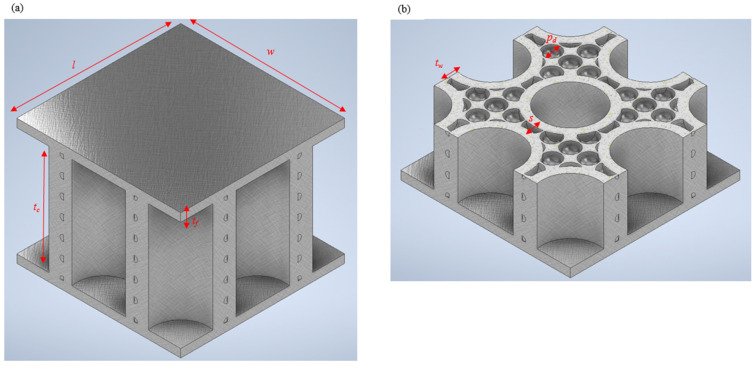
(**a**) CAD model and (**b**) geometric configuration of the biomimetic porous sandwich structure.

The biomimetic approach dictates that, when mimicking nature to create engineering designs, it is essential to keep the relative density of the designed structures (that is mimicked) close to the relative density of the natural mimicked source to have a fair comparison. As a result, we kept all of our samples close to a 0.6 relative density to claim the structures as “biomimetic”. We investigated the effect that the geometry of the biomimetic-designed samples had on their energy absorption capabilities by varying the thickness of the tube reinforcements and the spacing between the outer surfaces of the adjacent tubes. We intended to understand whether any geometric variations (while making sure that the relative density was close to 0.6) would affect the stiffness of the biomimetic structure. Hence, we did not introduce many variables with different relative densities (not the same as in nature), as this would divert the relative density away from the natural source of inspiration. The dimensions of the designed samples were based on ASTM C393 and are stated below in Table 1 and Table 2 along with a CAD model illustrated in Figure 2. The CAD models of the studied specimens were created using Autodesk Inventor. 

Taking specimen T1.5S3 as an example, the naming of the samples was mainly composed of two parts: (i) the thickness (*t_w_*) of the tubes (1.5 mm) and (ii) the spacing (*s*) between the outer surfaces of the two adjacent tubes (3 mm). 

An in-house fused deposition modelling (FDM)-based 3D printer, Ultimaker^TM^ S5 (Ultimaker, Zaltbommel, Netherlands) was used to fabricate the specimens. ABS filaments, supplied by Ultimaker^TM^, were chosen as the printing material in this study. ABS filaments can produce high-quality prints based on their roundness and consistent diameter. The manufacturer specifies these filaments to minimise warping and to ensure consistent interlayer adhesion. They are also tough and impact resistant, which are desirable characteristics for characterising energy absorption. It was reported that the maximum compressive force experienced by a cornstalk was 140 N at a displacement of 6 mm under compressive loading [33]. As a result, the mechanical properties of ABS were deemed sufficient for conducting a biomimetic structural study. The yield strength ($\sigma_{y}$) of ABS was 36 MPa, and it has a stiffness $\left(E\right)$ of 1.85 GPa. Other thermoplastic materials such as polylactic acid (a biodegradable polymer) have a much higher stiffness (3.25 GPa) and a higher yield strength ($\sigma_{y}$) (52.5 MPa) [42]. This makes PLA a more brittle material than ABS, which was not desirable in our study as the authors intended to learn about the deformation mechanisms of the designed structures. TPU (thermoplastic polyurethane), on the other hand, is known for its flexibility as it has a low stiffness (only 0.67 GPa) and negligible yield strength ($\sigma_{y}$), thus making it a ductile material and making it undesirable for studying deformation patterns. As such, ABS was chosen as the thermoplastic material for the following reasons:

- The convenience of printing smaller parts with minimal defects or warping effects;
- Its ability to identify all the properties needed for numerical modelling;
- The yield strength ($\sigma_{y}$) and the stiffness of ABS is suitable for studying the deformation of structures for energy absorption.

**Figure 3:**
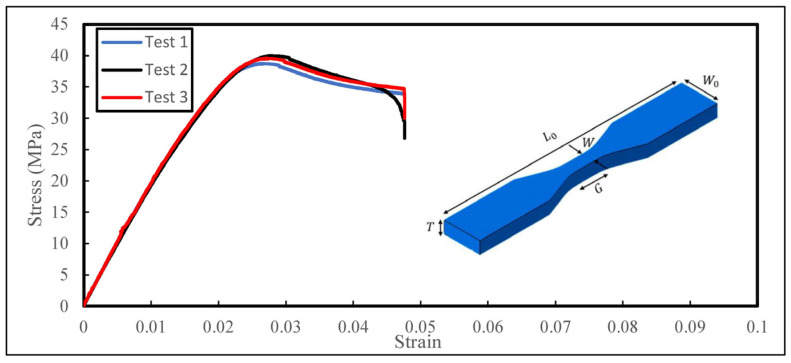
Stress–strain curves of 3D-printed tensile samples.

To determine the mechanical properties of the base material used to fabricate the biomimetic structures, the tensile properties of the printed samples were characterised. Tensile test samples were designed according to ASTM standard D638 [43]. A summary of the dimensions of the tensile samples are stated in Table 3. The average mechanical properties of the base material are summarised in Table 4. The tensile samples were printed horizontally on the print bed using the same settings as the biomimetic sandwich structure as shown in Table 5. A Shimadzu^®^ Universal Testing Machine (AG-X, Shimadzu Corporation, Japan) with a 100 kN load cell was employed in the experiment. Three samples were tested at a fixed loading rate of 1 mm/min. Figure 3 shows the stress–strain response of the tensile samples fabricated via 3D-printing technology.

The 3D printer was carefully calibrated to obtain the best quality printed specimens. Calibration is imperative to achieve an excellent final product. It programs the printer to extrude the right amount of ABS and allows for the precise rotation control of the print head. As for the printing parameters, optimised settings were taken into consideration from Gorana et al. [44]. The printing parameters are summarised in Table 5.

## 3. Methodology

### 3.1. Quasistatic Compression Test Design

Quasistatic compression tests were conducted on the printed biomimetic porous sandwich specimens along the uniaxial direction (parallel to the tubes) to investigate their compressive loading behaviour and energy absorption capability. The tests were conducted using Shimadzu^®^ Universal Testing Machine (AG-X) with a load capacity of 100 kN. Testing was performed using displacement control mode at a loading speed of 1.38 mm/min, which resulted in a strain rate of 0.001 s^−1^. All printed specimens were kept at room temperature inside a sealed bag for optimum storage conditions as suggested by the filaments supplier for at least 24 h before mechanical testing commenced. The loading curves were observed to determine the energy absorption during the compression process until 60% strain (densification region) of the full height of the specimen was reached.

### 3.2. Methodology for Damage Inspection

**Figure 4:**
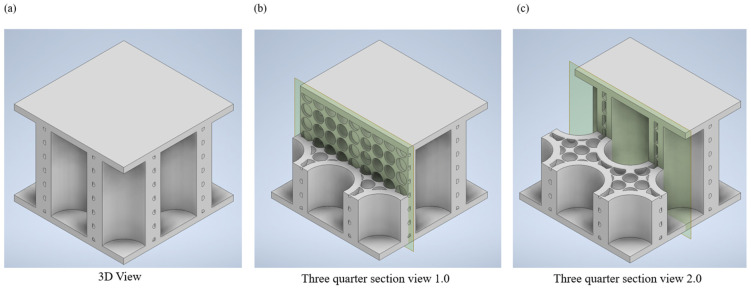
Two sections of interest for investigation of deformation patterns.

It is essential to analyse the structural deformation where the material has plastically deformed for energy absorption applications. The postmortem damage analysis of each specimen was conducted in three steps on two sections of the specimen as illustrated in Figure 4.

The three steps were:

- Cutting and grinding: The Buehler IsoMet Low Speed cutting machine was utilised to section the specimens precisely. Upon completing the cutting process, Tegramin from Struers was used to grind the sectioned specimens finely.
- Stereo microscopy: ZEISS SV8 stereo microscope was used to identify the defects and failures in the specimens during the compression process. A Canon SLR was used to inspect the damages in the specimens and to capture images.
- Optical profilometry: A full 3D scan of the damaged specimens was taken using NANOVEA Optical Profiler. The purpose of the profilometry was to profile surface morphology to perform a quantifiable analysis and to understand the surface roughness after cutting the sample using a diamond blade.

### 3.3. Finite Element Modelling

**Figure 5:**
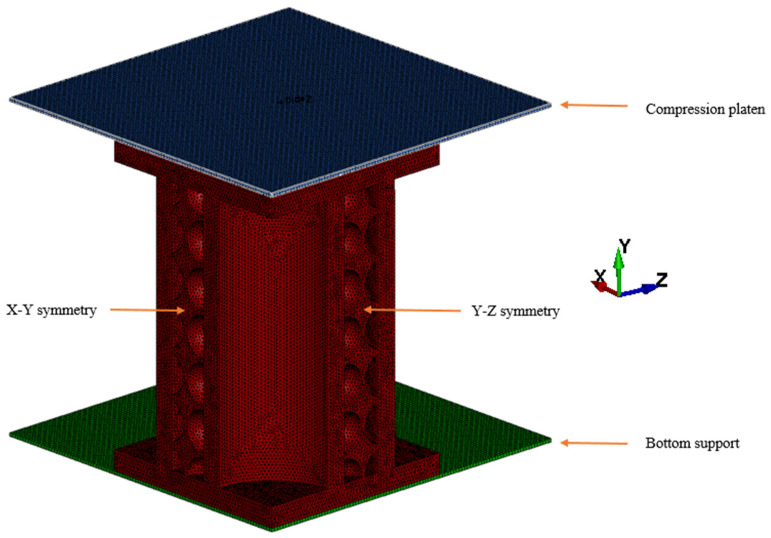
A quarter finite element of the compression plates and biomimetic porous sandwich structure.

A numerical model was developed using finite element explicit code LS-DYNA (LSTC, Livermore, CA, USA) to provide detailed information on the quasistatic response of the biomimetic structures. Some of the information collected was predictions of failure mechanisms, energy absorption capabilities, the evolution of the stress field, and validation of the experimental results. The model was built using the experimental setup. Only a quarter of the biomimetic porous sandwich structure, compression platen, and bottom support was created to improve the computational efficiency because of the symmetry of the setup as shown in Figure 5. The FEM method used here was previously successfully used by some of our authors, Sun et al. [45], to simulate the dynamic response of sandwich panels.

Since the sandwich structure incorporating various shapes and geometries would undergo deformation during the compression process, a small mesh size of 0.25 mm discretised the part. The adopted mesh size was verified to be adequate for producing converged results by a mesh sensitivity analysis. Solid elements were used to model the biomimetic structural part. 

The part was meshed using tetrahedral solid elements with 1 point tetrahedron (ELFORM = 10) to overcome negative volumes in large deformation and distortion situations. The degree of freedom (DOF) of the bottom support was all constrained to simulate the fixed boundary condition, and the symmetry boundary conditions were imposed on the symmetry of the sandwich structures. Using the example of the X-Y symmetry plane (from Figure 5), the symmetric conditions on that plane in FE model were applied as follows according to the LS-DYNA guidelines:

Boundary set conditions:

- Translational constraint in the local z-axis for degree for freedom (DOF)
- Rotational constraint in local x-axis for degree of freedom (DOF)
- Rotational constraint in local y-axis for degree of freedom (DOF)

The compression platen was only allowed to translate in the y-axis by restricting the DOFs of its nodes, except the y-translation node, and the initial velocities following those in the compression tests were applied to the compression platen. The contact between the plates and the sandwich structure was modelled with the automatic surface-to-surface algorithm. In addition, the interior contact treatment was applied using automatic self-contact to the solid elements of the core to keep them from merging or inverting. For high-strain modelling, automatic surface-to-surface conditions are a recommended contact type (according to LS-DYNA theory guidelines), as the orientation of parts relative to each other cannot always be anticipated because the model undergoes large deformation. The chosen contact treatment also checks for penetration on either side of the elements. During the experiment, dry surface conditions were used, and no visible slippage was observed. As such, the static coefficient of friction was applied in the contact treatment.

In the experiment, the deformation of the steel crosshead (100 kN) after tests was negligible. In the numerical simulation, therefore, the compression platen was assumed to be rigid and was modelled with Material Type 20 (*MAT_RIGID) with a density of 7.85 g/cm^3^, Young’s modulus of 210 GPa, and Poisson’s ratio of 0.3. The core was modelled with simplified Material Type 03 (*MAT_PLASTIC_KINEMATIC), which ignored the thermal effect and damage, with the ABS material properties listed in Table 4.

## 4. Results and Discussion

### 4.1. Experimental Results

**Figure 6:**
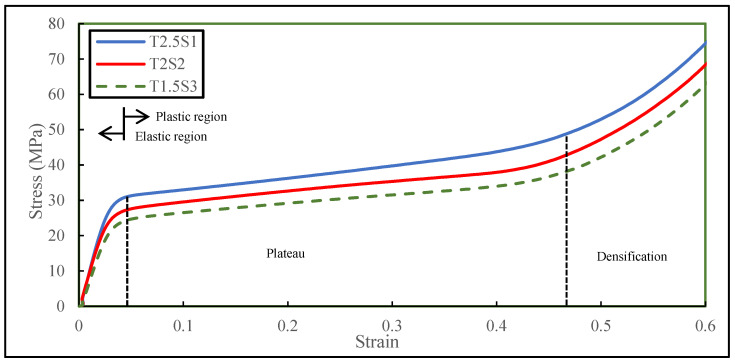
Stress–strain responses of the three biomimetic porous sandwich structure specimens.

The data collected from the experimentation of the three different specimens with similar relative densities were processed to generate the stress (σ)-strain (ε) response of the sandwich structure until a 60% strain level was achieved and until densification was observed (where the entire structure collapsed). The compressive response is shown in Figure 6. The compressive behaviour exhibits nonlinearity in the plastic region, implying that energy was dissipated during the process. Table 6 summarises the maximum compressive stressors exhibited by the specimens at the applied displacement during the test.

#### 4.1.1. Elastic Behaviour

The key parameters governing the elastic behaviour of any material are yield stress and elastic stiffness. A few interesting characteristics of quasistatic compressive behaviour can be observed in Figure 6. In the loading phase (elastic region), all the specimens with different tube thicknesses and spacings behaved linearly up to a compression of 1 mm. It denoted 4% of the initial specimen height. At this stage, no significant structural deformation was observed. It was noted that the yield stress (σ_y_) at a given strain increased linearly with an increase in the thickness of the tube in the matrix. Specimen T2.5S1 exhibited maximum yield stress, which was 13.3% greater than T2S2 and 29.1% greater than T1.5S1. On the other hand, Specimen T2.5S1 showed the highest stiffness compared to the other two specimens as shown in Table 7.

The experimental curves were obtained from a set of repetitive tests conducted as per ASTM standard C365. Error bars are presented in the table for the standard deviation of the results obtained. The errors obtained can be attributed to measurement uncertainty, instrument error, human error, and a compliance test. 

The results are presented as nominal values. We did not consider logarithmic strain in our study due to its limitations on strain measurements in large deformation mechanics. The structural analysis performed on the printed samples in this article did not consider the variation of the cross-section area of the elements or the buckling phenomenon. Hence, it is considered a research limitation. 

#### 4.1.2. Plastic Behaviour

For a given shape, the geometry and relative density of the biomimetic structures significantly affected the plateau slope. Specimen T2.5S1 was observed to have the largest plateau region compared to T2S2 and T1.5S3, as both of these specimens experienced an early densification strain (ε_d_) of 0.47 ± 0.006. In contrast, specimen T2.5S1 had a densification strain (ε_d_) of 0.52 ± 0.006 as summarised in Table 7. The densification strain (ε_d_) is the strain where the cells crush together and where the condensed material begins to strain. As the spacing between the tubes decreased, the samples could accommodate a higher portion of the tubes at the edges in a given volumetric fraction. This explains the underlying mechanism of the enhanced force resistance, stiffness, and larger plateau region of biomimetic specimen T2.5S1. Therefore, these structures exhibited more prolonged plateau stress after the onset of plastic deformation and large densification strain. On the contrary, specimens T2S2 and T1.5S3 had thinner tube walls and a higher number of pores in their volumetric fraction. More cell walls could deform and occupy the void spaces more systematically, enabling deformation to considerable strains yet not enabling strains larger than that of specimen T2.5S1.

Usually, the mechanical response of energy-absorbing structures maintains a reasonable constant stress over an extensive plastic strain regime. Therefore, the structures absorb large amounts of energy before reaching full densification to avoid excess stress transmission. Specimen T2.5S1, which had the thickest tube wall and the smallest spacing distance between the tubes, exhibited maximum stress (σ_m_) (74.3 ± 0.3 MPa) during the compression test at a 60% displacement limit. In comparison, specimen T2S2 exhibited a 7.8% lower maximum stress, and T1.5S3 exhibited a maximum stress that was 15.6% less than that of T2.5S1. The sandwich structure was nearly densified to a solid when the displacement reached 14 mm (i.e., 60% of the initial specimen height). In other words, the structural hierarchy was no longer effective beyond this point, implying that the specimen would nearly become a solid piece and that all the specimens were expected to behave similarly. 

Efficiency, η, is calculated from the energy absorption capacity normalised by the corresponding stress at any particular strain and is expressed by Equation (1):

(1) $$\eta \left(\varepsilon_{x}\right)=\frac{1}{\sigma_{x}}\int_{0}^{\varepsilon_{x}}\sigma \left(\varepsilon \right)d\varepsilon$$

where $\sigma_{x}$ is the corresponding stress for a particular strain, $\varepsilon_{x}$.

As stated in Table 7, specimens T2S2 and T1.5S3 exhibited lower densification strains than specimen T2.5S1. This was due to a decrease in tube thickness and an increase in the spaces between the tubes, which maximised the structural complexity of having more cell walls with voids within the specimen. Specimen T2.5S1 had less complexity within its structure, as it was mainly comprised of thick-walled tubes which underwent localised buckling and significant stress transmission during the compression as further explained in Section 4.2.2. It was observed that the effect of geometry on energy absorption and the plateau region was becoming more profound in a given relative density.

#### 4.1.3. Properties and Their Applications

One of the simple yet valuable applications of porous structures is to tune their mechanical properties, especially the effective stiffness of the structure. This can be achieved through local density variations or architecture modifications (thickening the tubes, changing spacing, changing pore size, or thickening the facesheets). It is a well-known fact that the porosity and stiffness of lightweight structures are interrelated. Similar findings were observed in our biomimetic structures. Tube thickness and spacing were tuned to identify which sample would generate a higher stiffness. A clear trend was seen in Figure 6 and Table 7 that, as the tubes were thickened, the sample stiffness increased gradually. Specimen T1.5S3, having the thinnest tube incorporated in its sandwich structure, was less stiff than the rest. As the thickness of the tube increased by 33.3% (2 mm), the stiffness of the sandwich structure increased by 23%. Furthermore, as the thickness of the tubes increased by 66.6% from the original, an overall increase in stiffness (E) by 34% (989.79 ± 1.70 MPa) was observed during compressive behaviour.

As the biomimetic specimens were demonstrated to have a larger plateau region with a near-constant load, they are more suited for crashworthiness applications, such as crash protection in vehicles, as a high energy absorption capability is the desired property for crashworthiness applications. However, one purpose of designing biomimetic structures is to serve lightweight applications. High stiffness and high specific energy absorption (SEA) are the prime desired properties for lightweight structural applications. Though specimen T2.5S1 was demonstrated to be stiffer among the three, based on the above findings, it is still unclear which specimen design is more suitable for lightweight applications as mass was not considered during the analysis. As a result, this is further discussed in Section 4.1.4 from the perspective of stiffness and weight to determine the applications suitable for the biomimetic designs.

#### 4.1.4. Energy Absorption Behaviour

In addition to the yield and plateau stress, the metric of greatest interest for comparative purposes was the specific energy absorption (SEA) for the proposed biomimetic structures inspired by the internal structure of a cornstalk, which is a measure of the amount of energy absorbed per unit mass (unit: kJ/kg) and which is expressed mathematically by Equation (2):

(2) $$\mathrm{SEA}=\frac{\int_{0}^{\varepsilon_{d}}\sigma d\varepsilon }{\rho }$$

where the numerator is the area under the stress–strain curve integrated into the densification strain (ε_d_) and where ρ represents the density of the biomimetic structures.

**Figure 7:**
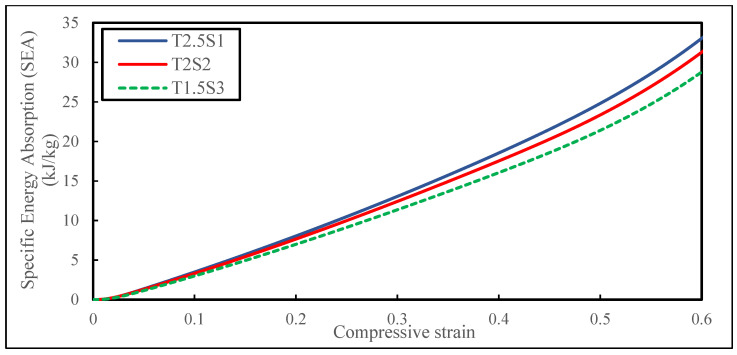
Comparison of SEA between biomimetic structures.

This measure could be arrived at by dividing the area under the force–displacement curve by the measured mass of the biomimetic structure under compression. It denotes the energy dissipated by the specimens due to the intermolecular dislocation in the strain-hardening process. The amount of SEA was calculated for each specimen design loaded up to 60% strain as summarised in Table 8 and illustrated in Figure 7.

It was identified that the biomimetic design specimen T2.5S1 outperformed the other specimen designs of the same sample size when compressed to 60% of the initial height. Specimen T1.5S3 could absorb 29.7 ± 0.05 kJ/kg during the overall compression stage until the densification region. The absorption capability increased by 7.4% (T2S2) as the thickness of the tube increased by 33.3% (2 mm), and the spacing between the tubes decreased by 33.3% (2 mm). Consecutively, the absorption capability further increased by 13.5% (T2.5S1) as the thickness increased by 66.6% (2.5 mm) from the original, and the spacing between the tubes decreased by 66.6% (1 mm). 

The data findings denoted that, as the mechanical properties of the specimens were tuned through relative density variation and architectural modifications, it significantly impacted the SEA characteristics of the biomimetic structures. It is equally important to point out that the mass of specimen T2.5S1 (15.7 ± 0.06 g) was 5.73% higher than specimen T2S2 (14.8 ± 0.06 g) of the same sample size, and it was 8.28% higher than specimen T1.5S3 (14.4 ± 0.06 g) of the same sample size. This suggests that weight should not be the only parameter taken into consideration for making improvements, as constantly lowering the weight can jeopardise the structural strength of the samples themselves. As a result, the relative density of the designed samples is an essential aspect to consider when designing. It should be noted that designing thinner tube walls significantly reduces weight and decreases the energy-absorbing capability as seen in the case of specimen T1.5S3. 

It was postulated that the stiffness and the amount of the energy absorption of the biomimetic structures (at a 60% relative density) would continue to increase when the mechanical properties were tuned through geometrical modification. However, further tuning the mechanical properties will gradually change the relative density of the biomimetic structure. As such, further investigation will be needed to understand how varying the geometry of each element of the specimen contributes to the stiffness and absorption capability at different relative densities, even though the relative density of the manufactured specimen would deviate away from the relative density of the cornstalk itself. 

**Figure 8:**
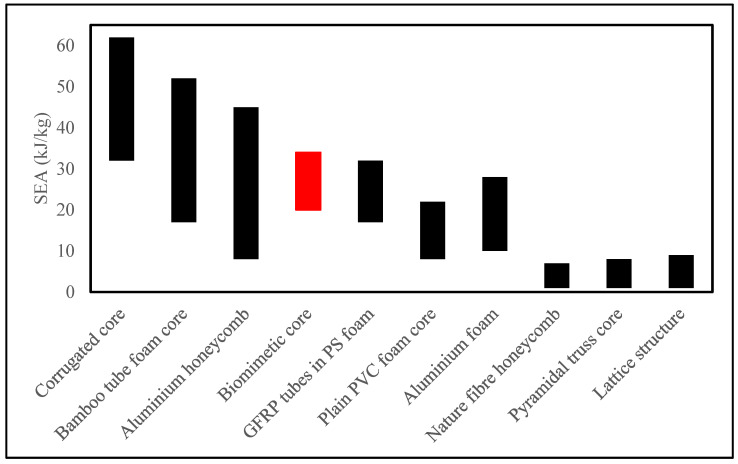
Comparison of energy absorption characteristics between various core structures, including the biomimetic design in this study (GFRP tubes in PS foam [46]; plain PVC foam core [46]; Lattice structure [47,48]; Pyramidal truss core [49]; Nature fibre honeycomb [50]; Aluminium foam [51]; Aluminium honeycomb [52]; Bamboo tube foam core [53]; Corrugated core [54]).

Lastly, Figure 8 above compares the quasistatic energy absorption capability of the studied biomimetic core with previously published data from tests on a wide range of core systems, including corrugated cores, aluminium foam, and honeycombs as well as truss and lattice structures [46,47,48,49,50,51,52,53,54] based on specific energy absorption. SEA allows for the comparison of the energy absorption capabilities of two different materials and geometries when the weight is an important parameter of the project [55]. The biomimetic structures compared very favourably to the other core materials. The biomimetic design (33.7 kJ/kg) was demonstrated to have a 17.6% superior energy-absorbing capability than the porous foam structure designed by Altenaiji et al. [51]; a 79.4% superior energy-absorbing capability than the porous honeycomb structure designed by Zuhri et al. [50]; and, lastly, a 73.5% superior energy-absorbing capability than the porous lattice structure designed by Mckown et al. [48]. However, the energy-absorbing capability of the corrugated structures designed by Rejab and Cantwell [54] surpassed the biomimetic design proposed in this study. This suggests that the proposed designs offer the potential for use in designing lightweight energy-absorbing structures. It also depends on the type of applications, such as crashworthiness applications, lightweight structures, support structures, or shock absorption, since other core materials also have greater or lower energy-absorbing capabilities.

#### 4.1.5. Postmortem Damage Analysis

**Figure 9:**
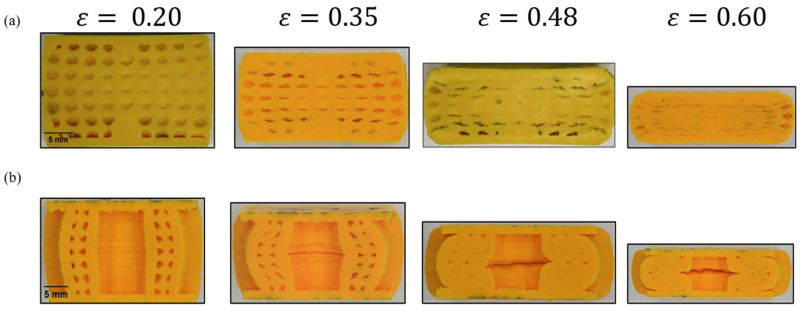
Deformation of the biomimetic specimen after cutting and grinding. (**a**) Compressive behaviour of the pores. (**b**) Compressive behaviour of the tubes.

The biomimetic structures showed significant localised stress from the beginning of the compression. Deformation initiated at the comparatively weaker cell walls of the pores located in the middle (perpendicular to the compression axis) of the specimens along the walls of the inner tube with increasing strain as shown in Figure 9a,b at ε=0.35.

**Figure 10:**
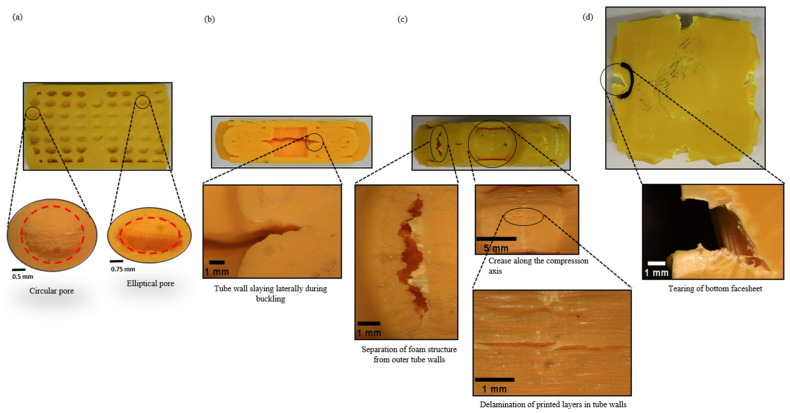
Identified failure mechanisms during energy absorption process captured using stereo microscopy. (**a**) Buckling of pores leading to a change in their shapes. (**b**) Splaying of the inner tube during buckling. (**c**) Separation and delamination of tube walls. (**d**) Tearing occurrence on facesheets at ε=0.60.

As the top edges of the pores moved towards the bottom edges, the pores subsequently changed their shape from circular to elliptical geometry as shown in Figure 10a. The cell wall collapsed plastically, and the stress propagated to the following weaker zones (towards the top and bottom). As a result, it was noted that not all the pores deformed in the same region simultaneously. This sequential plastic deformation process delayed densification to the effect and produced a comparatively better plastic plateau and energy absorption efficiency. Some of the pores at the edges were left undeformed, as they were “out-of-the-sample” due to the buckling effect as shown in Figure 9a at ε=0.48 and ε=0.60. As a result, the force exerted during the compression stage by the facesheets on the core did not affect the pores at the edges. As such, plastic deformation occurred discontinuously. This produced multiple irregular shapes in the voids and stress distribution during uniaxial compression due to the strength inhomogeneity throughout the structure. It was observed that the vertical cell walls of the pores carried more stress than the cell walls oriented perpendicular to the loading axis, as the cell walls aligned with the loading axis underwent compression at the beginning and subsequently experienced buckling at large strains. This implies that the vertical cell walls were more responsible for enhancing the plastic stress during compression.

An examination of the failed tubes at the plateau regime indicated that the crushing process led to the middle region of the inner cylinder splaying outwards as shown Figure 9b at ε=0.35 and ε=0.48. No fine fragments or debris occurring from the tube walls were observed as tube walls tended to have excellent performance of axial loading resistance. The outer tube walls were deformed, and the buckling phenomenon was observed, forming a C-shaped wall structure. In this biomimetic structure, though the tube wall exhibited the most significant contribution to energy absorption, the foam surrounding the tube walls constrained the splaying process, resulting in different deformation behaviours between the inner and outer tubes as it was crushed and subsequently disintegrated. The failure process also indicated the high level of constraint applied by the foam on the tubes to crush along its longitudinal axis. It explains the enhancing effect of the foam in terms of overall energy absorption. More so, the degree of interaction with the skin of the sandwich structure during the process resulted in a higher plateau force during the crushing process, which translated to better energy absorption.

As the barrelling phenomenon continued, the localised strain was observed in the middle of the inner tube as the expansion propagated laterally as illustrated in Figure 10b. In contrast, the outer tubes developed creases at the centre of the tube wall parallel to the compression axis as shown in Figure 10c. The outer tubes failed due to the delamination of the printed layers in the tube walls, buckling, and crack initiation, leading to the separation of the foam core from the tube walls embedded in the matrix. Such failure suggests that a significant amount of energy was absorbed during the compression process.

**Figure 11:**
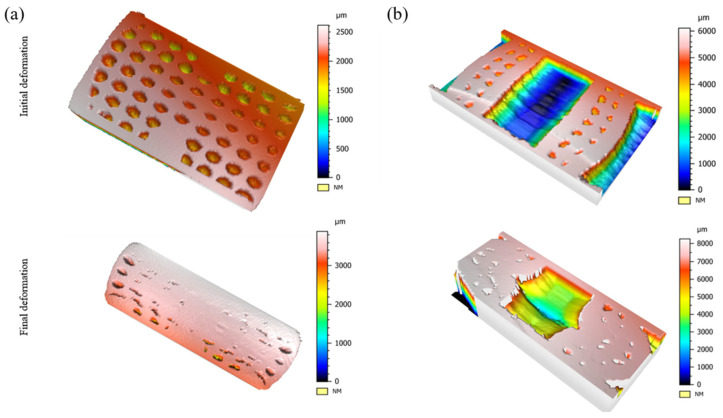
Surface morphology of the deformed specimens produced through optical profilometry. (**a**) Pores. (**b**) Tubes.

A quick observation of the surface morphology in Figure 11 indicates that the surface roughness achieved from the cutting process at both sections of the specimens was reasonably acceptable to determine the profiles of the tubes and pores at different strain levels. Focusing on the contour plots in Figure 11a, it was noted that the pores located away from the centre and at the top and bottom edges of the specimen were deeper (1.5 mm). In contrast, the voids demonstrated a depth reduction of 66.6% (0.5 mm) at the centre. These findings are understandable as the pores at the centre were surrounded by the tube walls and assisted the splaying process of the tubes when buckling in the middle perpendicular to the compression axis. The morphology in Figure 11 also supports the statement that, during initial deformation, the weaker cell walls of the pores at the middle origin were the first to undergo plastic deformation. In addition, the geometry of the circular and elliptical pores was determined from Figure 10a and Figure 11a. It was stated that the area of the voids increased by 35% during the buckling and shape-changing processes at the initial deformation stage and was expected to further increase gradually as the vertical cell walls underwent permanent deformation throughout compression.

On the other hand, it was interesting to see in Figure 11b that the depth of the tube at the centroid increased locally during plastic deformation. Analysing the contour plots dictated that the depth of the tube during the buckling process increased by 40%. In other words, the diameter of the tube in the middle section perpendicular to the compression axis increased by 40% as the diameter increased from 5 mm to 7 mm. The morphology collected from initial to final deformation in Figure 11 suggests that, during the plastic densification stage, the pores were entirely collapsed (as no significant visible depths were seen) and were ineffective in providing any further support to resist the splaying process at ε = 0.6 during the compression stage.

Lastly, the facesheets were observed to expand plastically during the compression process until 60% strain was reached, and the compression test was stopped. As more significant efforts were required to collapse the pores, deform the tubes, and expand the facesheets, a rapid increase in steepness was demonstrated by the rapid increase in the stress experienced in the densification region. For specimen T2.5S1, it was observed that, as the bottom facesheet expanded plastically, it started failing at the centre of all four edges as shown in Figure 10d.

### 4.2. Numerical Results

#### 4.2.1. Validation of the FE Model

The finite element simulations of the compression tests were conducted using 3D CAD geometry to manufacture the experimental specimens to validate the numerical simulation using the same experimental conditions. The numerical simulation could extract the contact force between the compression platen and the sandwich structure. The displacement data are available from the translation movement of the rigid compression platen. Therefore, the simulation results were compared with the experimental data in terms of the stress–strain response, energy absorption capability, and structural collapse mechanisms to show the reproducibility of the modelling approach.

**Figure 12:**
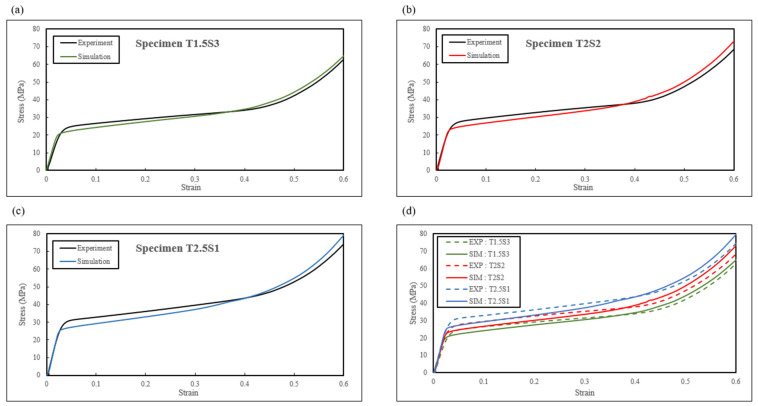
Comparison of experimental and simulated stress–strain curves of (**a**) specimen T1.5S3, (**b**) specimen T2S2, and (**c**) specimen T2.5S1. (**d**) Comparison of the stiffness of all three specimens.

**Figure 13:**
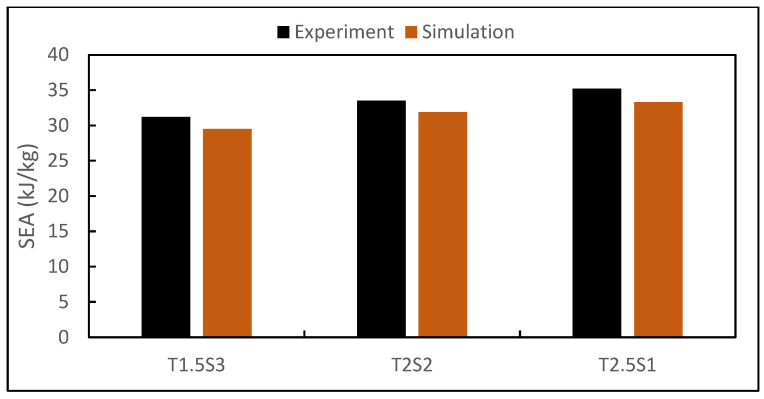
Comparison of experimental and simulated specific energy absorption (SEA) at ε=0.60.

Figure 12 compares the stress–stress response up to ε=0.60 of the three biomimetic structures. It is evident that the trends of the simulated stress–strain response were in good agreement with the experimental results. However, it was observed that there was a slight discrepancy in simulated yield stress (σ_s_), as it was slightly lower than the experimental data. With increasing strain, local deformation occurred in the pores and tube walls, leading to a drop in the max stress (σ_m_) in the experimental data. The simulation results systematically overestimated the maximum experimental stress (σ_m_), which is a common occurrence as stated in previously studied additively manufactured samples [56,57,58]. As for energy absorption, Figure 13 compares the specific energy absorption capabilities obtained from the experimentation and simulation of the biomimetic structures. There was a good consistency between the bars from the experiments and simulations, and the errors of the specific energy absorption values were less than 6% as summarised and compared in Figure 9.

Some of the assumptions taken into account when modelling were as follows:

- The simulations of the compression test were run with a perfectly plastic assumption. In reality, localised defects are inevitable due to the 3D printing process. Warping and irregular surface features were some of the printed parts’ defects.
- The structure was assumed to be isotropic, i.e., a uniform base material property throughout the structure. However, this analytical model did not consider material anisotropy due to the 3D printing process.
- The structure was assumed to have a uniform relative density during modelling. However, the manufactured samples might not have had a uniform relative density due to microporosity in the printed layers.
- Given that the geometry of the designed specimens was symmetric and that force distribution was even, the quarter-symmetrical model was adopted to run the simulation reasonably, assuming the behaviour would be the same as the full model.
- The deformation of the tetrahedral solid elements underwent large distortion when the strain approached 30% and above. Two types of contact parameters were used to overcome the negative volume, and one point tetrahedron (ELFORM 10) was chosen. It was assumed that all of the elements in the sample would not have any negative volume, which could affect the numerical results.
- The compression plate was assumed to be rigid, and the material model was chosen accordingly.

The discrepancy between the experimental and numerical modelling results, as shown in Table 9, Figure 12 and Figure 13, was contributed to the assumptions stated above, leading to variations in the experimental results.

**Figure 14:**
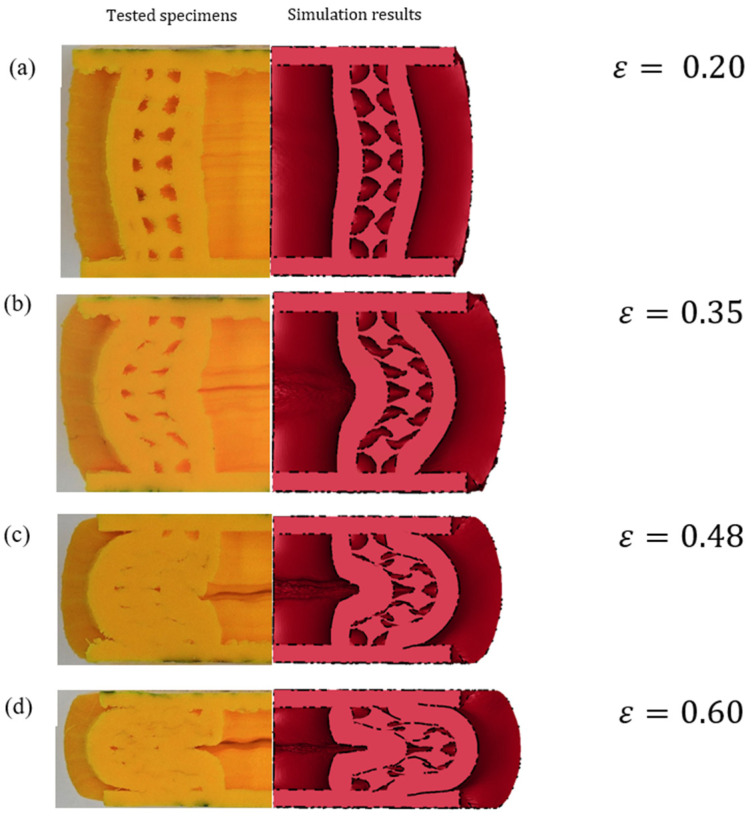
Experimental and simulated failure mechanisms at different strain levels during compression at (**a**) ε=0.20, (**b**) ε=0.35, (**c**) ε=0.48, and (**d**) ε=0.60.

Figure 14 illustrates the cross-sectional view showing the deformation characteristics of the sandwich structure at different strains during compression. As the deformation modes of the three biomimetic structures were similar, only one type (specimen T1.5S3) is illustrated in Figure 14 for comparison purposes. It can be seen that the simulated local deformation of the inner tube demonstrated behaviour similar to the experimental observations. The numerical and experimental results demonstrated the similar buckling behaviour of the core at different strain levels during compression until ε=0.60. The maximum simulated SEA obtained when the structure was compressed to 60% of the original height was less than 6% of the experimental SEA. The errors were minor, and the maximum error was only 5.45%. Based on the above analysis, it was confirmed that the developed finite element model could produce accurate and reliable results regarding the stress–strain response, energy absorption, and the deformation pattern of the biomimetic structures. 

#### 4.2.2. Deformation Mechanism

**Figure 15:**
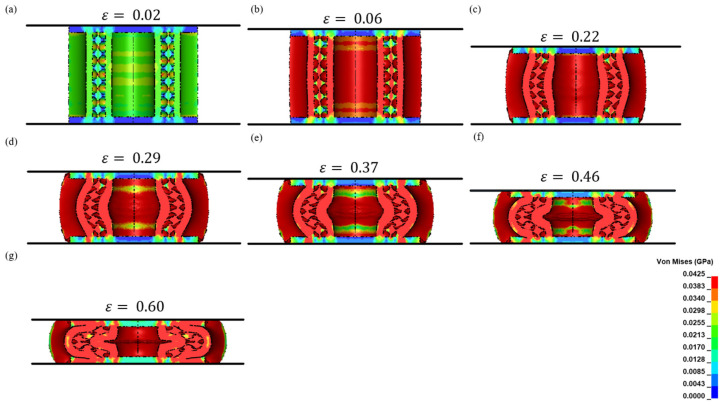
Stress transmission within the structure at different strain levels during compression (**a**) ε=0.02 (**b**) ε=0.06 (**c**) ε=0.22 (**d**) ε=0.29 (**e**) ε=0.37 (**f**) ε=0.46 (**g**) ε=0.60.

Figure 15 demonstrates the compressive behaviour of the biomimetic sandwich structure until a displacement of 14 mm was reached, respectively. In this case, the deformation of the structure recorded in the experiment was well captured by the simulation. 

At the beginning of compression, an elevated-stress region was experienced by the core itself (green V.M contour) as demonstrated in Figure 15a. At ε=0.02, the maximum stress (σ_m_) encountered by the core was 18.5 ± 0.3 MPa as shown in Figure 6, and it was within the elastic limit. However, as the compression stage progressed to ε=0.06, the vertical cell walls of the pores had a higher stress concentration (red V.M contour) than the horizontal cell walls as shown in Figure 15b. At this stage, the applied force was transmitted to the vertical cell walls of the pores and the tube walls, causing the structure to reach a maximum stress (σ_m_) of 22.7 ± 0.3 MPa, while the maximum stress (σ_m_) of the horizontal cell walls was much lower. That is, deformation initiated in the vertical cell walls due to compressive stress, whereas the horizontal walls experienced minor tensile stress and remained within the elastic limit. 

Additionally, the top and bottom regions of the core at ε=0.06 had a lower stress region (orange V.M contour) than the central axis perpendicular to the direction of the compression axis. This was the underlying cause of the buckling effect of the sandwich core. The stress experienced at this stage was beyond the elastic limit. As a result, the structure began to deform plastically from this stage onwards. 

As the buckling phenomenon progressed to ε=0.22, the majority of the structures within the sandwich matrix was observed to have a high level of stress (28.2 ± 0.3 MPa), except the horizontal cell walls of the pores that were located only in the top and bottom regions, as shown in Figure 15c. At this stage, the plastic deformation of the change in the shape of the pores produced multiple irregular shapes in the voids and stress distribution while the stress propagated to the weaker zones. It was also observed that the maximum stress (σ_m_) (22.2 ± 0.3 MPa) on the tube walls and vertical walls of the pores was significantly lower than the yield stress (σ_y_) (37 MPa) of the base material. In addition, there was no high-stress region observed in the facesheets of the sandwich structure.

Figure 15d,e demonstrates that the top and bottom regions of the sandwich structure remained slightly unaffected (green V.M contour) by the compressive force. A maximum stress (σ_m_) of 33.9 ± 0.3 MPa was concentrated at the centre of the structure, resulting in the propagation of the splaying process of the inner tube laterally. In contrast, the outer tube formed a C-shaped wall undergoing plastic deformation after the initial yielding of the structure. 

The independency of the top and bottom regions was first discovered once the samples were sectioned at different strains, and the deformation patterns were identified as shown in Figure 9a. More of the cell walls of the pores collapsed in the midsection region. To understand the underlying hypothesis of this deformation behaviour, we opted to perform numerical modelling to identify the stress field distribution during the compression process. During the plateau stage between ε=0.06 and ε=0.46, it was observed that the highest stress region was at the center of the sandwich structure as shown in Figure 15. Hence, this led the inner tube wall to buckle and change shape. It was noted in numerical modelling that the independence of the top and bottom regions lasted until the densification stage was reached. From this stage, the stress concentration was even throughout the structure as shown in Figure 15g.

Lastly, it was observed in Figure 15f,g that, as compression reached ε=0.60, the stress zone was evenly distributed and that the crushed cell walls of the pores filled the voids within the structure. The structure was ineffective in providing any further support and exceeded the yield stress (σ_y_) of the base material as a maximum stress (σ_m_) of 62.7 ± 0.3 MPa was achieved as shown in Figure 6. Therefore, the deformation propagation observed in the experiments during compression agreed well with the corresponding Von Mises stress contours at seven different strains as shown in Figure 15.

Overall, the purpose of numerical modelling was as follows:

- To predict the failure/deformation behaviour of the tubes and pores embedded in the biomimetic structure as modelling ran with a perfectly plastic assumption. The deformation pattern identified during the experiment could be different from that identified during numerical modelling due to defects in the samples or any possible human errors while conducting the experiments. In the end, it was noted that the deformation pattern observed during the experiments was acceptable as it was well matched with that observed during modelling.
- To understand the underlying hypothesis of the deformation behaviour through stress field distribution. This adds quantitative physical data on the deformation mechanisms which authors cannot collect solely from the experiment during the deformation process.
- To obtain the stress threshold for buckling, which can be determined through simulations to provide quantitative limits on the strain range for the given structure. In our study, the stress threshold for buckling was determined to be 21.3 MPa–42.5 MPa on a strain regime between $\left(\varepsilon \right)$ 0.02 and 0.29.

- To obtain stress contours that could be used to design better geometries for specific applications. In numerical modelling, variations can be made at relatively low costs and can be simulated rather than being made through the expense of 3D printing and being made by conducting experiments.
- To perform data validation on the stress–strain responses of the experimental and modelling results. Visualising the trendline (as shown in Figure 12) provided the authors with the justifiability of the accuracy of the printed samples and experiments.

## 5. Conclusions

A novel bioinspired porous sandwich structure was proposed by mimicking the internal structure of a cornstalk on a meso scale. ABS filaments were used due to their toughness, and the compressive responses of three biomimetic structures at quasi-static strain rates were evaluated. In addition, we conducted a detailed local stress and deformation analysis in our finite element simulations and correlated the deformation modes with overall mechanical behaviour.

The naming of the samples (e.g., T1.5S3) was mainly composed of two parts: (i) the thickness of the tubes (1.5 mm) and (ii) the spacing between the outer surfaces of the adjacent tubes (3 mm). Based on the results, the main conclusions were as follows:

- Specimen T1.5S3, which had the thinnest tubes in the matrix, was less stiff (737.51 ± 1.94 MPa) than the other designs. It was noted that, as the thickness of the tube increased by 33.3%, the stiffness of the biomimetic structure increased by 23%.
- Regarding SEA, the biomimetic-designed specimen outperformed the other types of cores, such as foams, honeycombs, lattices, and truss cores. This suggests that the proposed designs offer the potential for use in designing lightweight energy-absorbing structures.
- The outer tubes failed due to the delamination of the layers in the tube walls, buckling, and crack initiation, leading to the separation of the foam core from the tube walls embedded within the matrix. Such a failure suggests that a significant amount of energy was absorbed during the compression process. The inner tube walls splayed outwards in the lateral direction during the crushing process. Localised stress was observed at the centre (perpendicular to the compressive axis) of the inner tube as the expansion propagated.
- The geometry of the pores changed and deformed plastically during the crushing process. It was noted that the vertical cell walls were more responsible for enhancing the plastic response during compression.
- The developed finite element model produced accurate and reliable results regarding the biomimetic structures’ stress–strain response, energy absorption, and deformation pattern with less than a 6% error. The difference in the results was not significantly large.

As well as identifying how the structure deformed and failed during the compression process, this study has shown the potential for superior performance in energy-absorbing applications of cornstalk-inspired lightweight porous structures. 

## Figures and Tables

**Table 1:** Geometrical parameters of the sandwich structures (units: mm).

	Length (*l*)	Width (*w*)	Core Thickness (*t_c_*)	Facesheet Thickness (*t_f_*)	Total Thickness (*t*)
**Dimensions**	30	30	20	1.5	23

**Table 2:** Specifications of the biomimetic structures (units: mm and g).

Specimen ID	Wall Thickness (*t_w_*)	Outer Diameter (*d_o_*)	Inner Diameter (*d_i_*)	Spacing (*s*)	Pore Diameter (*p_d_*)	Mass	Relative Density, ρ
T1.5S3	1.5	13	10	3	3	14.8	0.58
T2S2	2	14	10	2	3	15.1	0.62
T2.5S1	2.5	15	10	1	3	16.2	0.65

**Table 3:** Dimensions of tensile samples (units: mm).

	Length $\left(L_{0}\right)$	Width of Narrow Section (W)	Gauge Length (G)	Width Overall $\left(W_{0}\right)$	Thickness (T)
**Dimensions**	115	6	25	19	4

**Table 4:** Input material parameters of acrylonitrile butadiene styrene (ABS).

Properties	ABS
Density (g/cm^3^)	1068
Young’s modulus (GPa)	1.85
Yield strength (MPa)	36
Ultimate strength (MPa)	40
Poisson’s ratio	0.35
Failure strain at break	0.047
Hardness (shore D)	76

**Table 5:** Three-dimensional printing parameters.

Printing Conditions	Parameters
Layer height (mm)	0.1
Infill (%)	100
Printing speed (mm/s)	55
Size of nozzle head (mm)	0.25
Nozzle head temperature °C	250
Build platform temperature °C	85
Brim (mm)	4

**Table 6:** Variation in compressive responses.

	Maximum Compressive Stress (MPa)		
Displacement Limit	T2.5S1	T2S2	T1.5S3
1 mm (4%) Elastic	30.6 ± 0.3	27.0 ± 0.3	23.7 ± 0.3
10 mm (43%) Plateau	43.5 ± 0.3	37.8 ± 0.3	33.8 ± 0.3
14 mm (60%) Densification	74.3 ± 0.3	68.6 ± 0.3	62.7 ± 0.3

**Table 7:** Mechanical properties of the biomimetic structures.

	Specimen		
	T2.5S1	T2S2	T1.5S3
**Stiffness (MPa)**	989.79 ± 1.70	914.01 ± 1.55	737.51 ± 1.94
**Densification Strain ($\varepsilon_{d}$)**	0.52 ± 0.006	0.47 ± 0.006	0.47 ± 0.006

**Table 8:** Energy absorption capabilities.

	Specimens		
	T2.5S1	T2S2	T1.5S3
SEA (kJ/kg)	33.7 ± 0.05	31.9 ± 0.05	29.7 ± 0.05
Mass (g)	15.7 ± 0.06	14.8 ± 0.06	14.4 ± 0.06

**Table 9:** Comparison of experimental and numerical results.

	Specific Energy Absorption (SEA)		
Specimen	Experimental Data (kJ/kg)	Simulation Data (kJ/kg)	Error (%)
T1.5S3	31.2	29.5	−5.45%
T2S2	33.5	31.9	−4.78%
T2.5S1	35.2	33.3	−5.40%
